# Synergistic In Vitro Anticancer Toxicity of Pulsed Electric Fields and Glutathione

**DOI:** 10.3390/ijms232314772

**Published:** 2022-11-25

**Authors:** Christina M. Wolff, Sander Bekeschus

**Affiliations:** ZIK Plasmatis, Leibniz Institute for Plasma Science and Technology (INP), Felix-Hausdorff-Str. 2, 17489 Greifswald, Germany

**Keywords:** apoptosis, electrochemotherapy, ECT, GSH, leukemia, melanoma, reactive oxygen species, skin cancer, SCC, squamous cell carcinoma

## Abstract

Despite continuous advancement in skin cancer therapy, the disease is still fatal in many patients, demonstrating the need to improve existing therapies, such as electrochemotherapy (ECT). ECT can be applied in the palliative or curative setting and is based on the application of pulsed electric fields (PEF), which by themselves exerts none to low cancer toxicity but become potently toxic when combined with low-dosed chemotherapeutics such as bleomycin and cisplatin. Albeit their favorable side-effect profiles, not all patients respond to standard ECT, and some responders experience tumor recurrence. To identify potential adjuvant or alternative agents to standard electrochemotherapy, we explored the possibility of combining PEF with a physiological compound, glutathione (GSH), to amplify anticancer toxicity. GSH is an endogenous antioxidant and is available as a dietary supplement. Surprisingly, neither GSH nor PEF mono treatment but GSH + PEF combination treatment exerted strong cytotoxic effects and declined metabolic activity in four skin cancer cell lines in vitro. The potential applicability to other tumor cells was verified by corroborating results in two leukemia cell lines. Strikingly, GSH + PEF treatment did not immediately increase intracellular GSH levels, while levels 24 h following treatment were enhanced. Similar tendencies were made for intracellular reactive oxygen species (ROS) levels, while extracellular ROS increased following combination treatment. ROS levels and the degree of cytotoxicity could be partially reversed by pre-incubating cells with the NADPH-oxidase (NOX) inhibitor diphenyleneiodonium (DPI) and the H_2_O_2_-degrading enzyme catalase. Collectively, our findings suggest a promising new “endogenous” drug to be combined with PEF for future anticancer research approaches.

## 1. Introduction

Despite continuous advancement in skin cancer therapy, the disease is still fatal in many patients [1]. Typical types of skin cancer with high malignancy are malignant melanomas and squamous cell carcinomas (SCC), including those deriving from head and neck tissue (HNSCC). HNSCC originates from mucosal epithelial tissues of the mouth, throat, salivary glands, nose, and sinuses. Etiologically, excessive alcohol and tobacco use and human papillomavirus infection are mainly linked to HNSCC prevalence. The five-year survival rate (incidence: 650,000 globally) is 67% (2013), substantiating the need for further improving therapies. With a 5-year survival rate of only 20%, malignant metastatic melanoma provides similar challenges. Therapeutic strategies depend on the tumor location, cancer stage, patient’s age, and general health. Traditional skin cancer therapy includes surgery, radiotherapy, chemotherapy, targeted therapy, immunotherapy, or combinations of those within multimodal schemes [2,3].

Electrochemotherapy (ECT) is among the canon of skin anticancer therapies [4]. Due to many other effective therapies and the large number of patients failing these therapies, ECT is primarily applied in the palliative setting, albeit curative approaches have been described [5]. Bleomycin and cisplatin are primarily used chemotherapeutics applied within ECT schemes and show overall good tolerability as they are administered at low doses [6,7]. Notwithstanding, ECT’s response rates in treating skin cancer patients remain to be improved [8]. Accordingly, several approaches have been reported in the past focusing on novel agents or physical treatment modalities to be potentially combined with PEF, such as doxorubicin [9], 17β-Estradiol [10], supraphysiological calcium [11], gas plasma technology [12], and checkpoint antibody immunotherapies [13].

This study aimed to investigate the potential of an endogenous compound, glutathione (GSH), to combine with PEF for promoting cytotoxic responses in squamous cell carcinoma and malignant melanoma cells in vitro. Glutathione is the most critical human antioxidant and has also been attributed a role in cancer and the success of onco-therapies [14]. Single PEF and GSH treatment did not exert cytotoxic effects, while combining both dramatically increased cell death responses up to 50-fold, partially in a reactive oxygen species (ROS)-dependent fashion.

## 2. Results

### 2.1. Synergistic GSH and PEF Treatment Shows Striking Anticancer Toxicity

**Figure 1:**
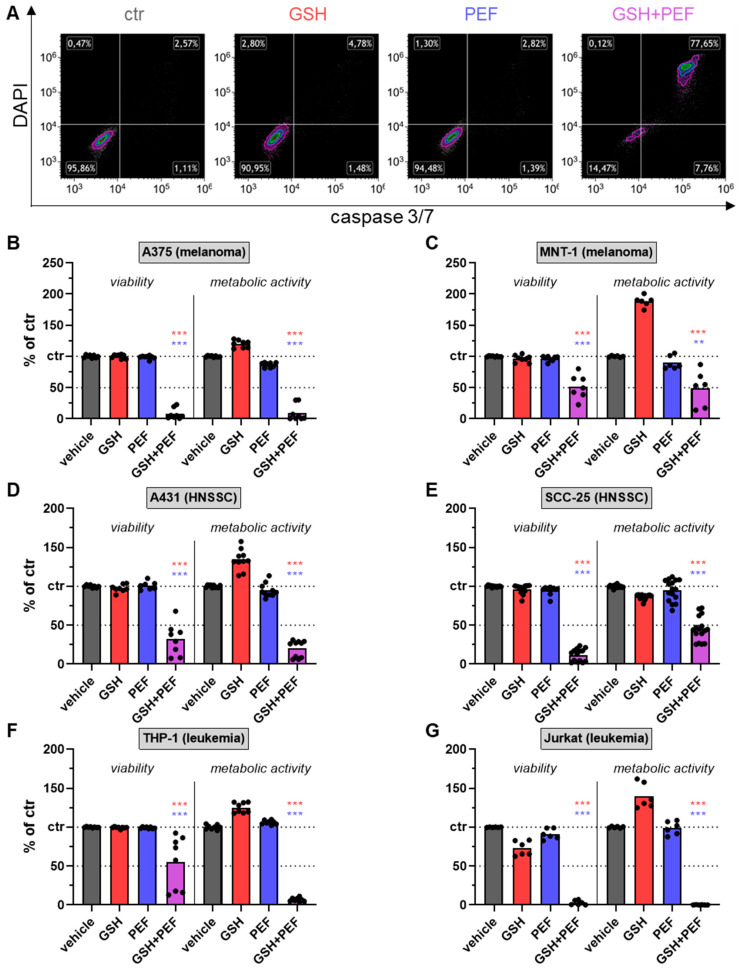
Reduced metabolic activity and viability following combined GSH and PEF treatment. (**A**) representative density plots of active caspase 3/7 fluorescence and DAPI in untreated, GSH-treated, PEF-treated, and GSH + PEF-treated A431 cells; (**B**–**G**) viability (left sides) as determined as percentage of cells negative for caspase 3/7 fluorescence and DAPI and metabolic activity (right sides) as determined using a resazurin-based microplate reader assay of vehicle, mono-, or combination-treated A375 (**B**), MNT-1 (**C**), A431 (**D**), SCC-25 (**E**), THP-1 (**F**), and Jurkat (**G**) cells after data normalization to vehicle conditions. Data show one representative (**A**) or mean of three independent experiments with several replicates each. Statistical analysis was performed using one-way analysis of variances with Turkey’s multiple comparison test (**: *p* < 0.01, and ***: *p* < 0.001; colors indicate significant combination comparison to either GSH or PEF).

**Figure A1:**
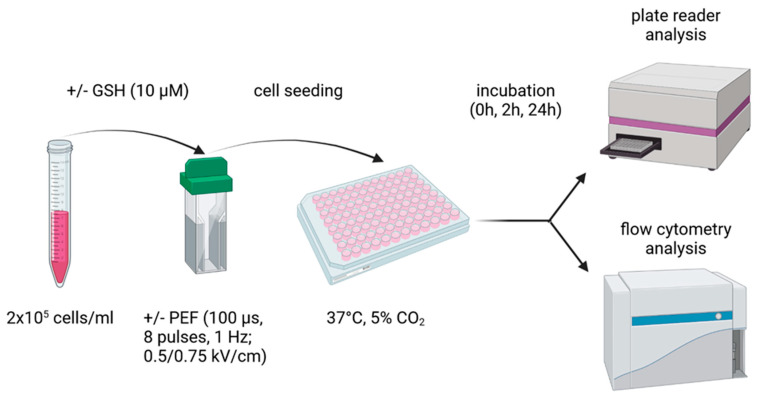
Study design. Tumor cells in the presence or absence of glutathione (GSH) were exposed to pulsed electric fields (PEF) prior to seeding in microwell plates, incubation, and downstream analysis using flow cytometry and plate reader-based assays.

The purpose of this study was, based on preliminary findings, to investigate whether PEF treatment together with GSH exposure would combine to have cytotoxic effects. To this end, mono and combination treatments were performed, and several cell types were analyzed using a microplate reader and flow cytometry assays (Figure A1). Four skin cancer cell lines (with A375 and MNT-1 being malignant melanoma cells and A431 and SSC-25 being squamous cell carcinoma cells) were used to address this question. In untreated, GSH-exposed, and PEF-treated cells investigated at 24 h, the number of dead or dying cells was virtually unchanged, while combination treatment potently induced terminal cell death (Figure 1A, viability). Similar results were obtained when considering the metabolic activity. Quantification of these results showed a dramatic (50-fold) GSH + PEF combined toxicity in A375 cells (Figure 1B). In MNT-1 cells, results were less pronounced but still very potent (Figure 1C). The combined effect was more pronounced in A431 (Figure 1D) and SCC-25 (Figure 1E) cells. In all four skin cancer cell lines, the results of viability testing using flow cytometry and metabolic activity testing using a resazurin-based assay matched, except for SCC-25 cells. In the latter, metabolic activity was higher, indicating a subset of metabolically very active cells surviving the treatment. In all four skin cancer cell lines tested, the exposure to either GSH or PEF alone did not induce cell death, demonstrating the potency of the combination treatment. To assess whether similar results would be achieved in other cancer cell lines, the experiments were performed in THP-1 (Figure 1F) and Jurkat (Figure 1G) leukemia cells. A highly significant synergistic effect was observed in both cases, particularly strong in Jurkat cells. Coefficient of drug interaction (CDI) calculations showed outstanding synergistic effects of the PEF and GSH combination treatment across six cancer cell lines (Table 1).

### 2.2. Presence and Roles of Reactive Oxygen Species in Combined GSH + PEF Toxicity

**Figure 2:**
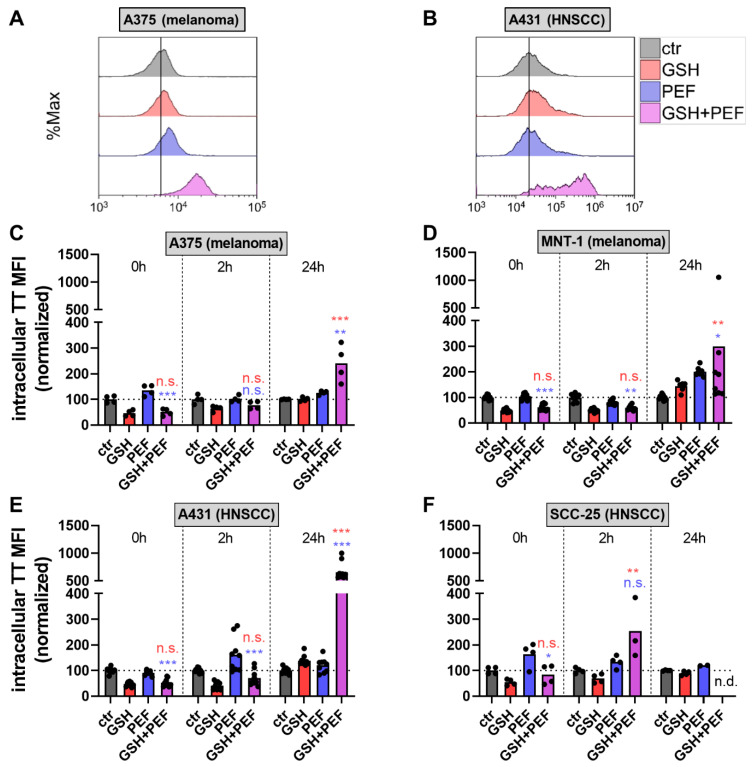
Intracellular GSH levels. (**A**,**B**) representative overlay histogram of intracellular thioltracker (TT) violet fluorescence in A375 (**A**) and A431 (**B**) cells across four treatment conditions as determined using flow cytometry; (**C**–**F**) intracellular TT fluorescence of vehicle, mono-, or combination-treated A375 (**C**), MNT-1 (**D**), A431 (**E**), and SCC-25 (**F**) cells freshly stained and investigated immediately (0 h), 2 h, and 24 h post-treatment, and subsequent data normalization to control (ctr) conditions. Data show one representative (**A**,**B**) or mean of at least two independent experiments with several replicates (dots). Statistical analysis was performed using one-way anova with Turkey’s multiple comparison test (*: *p* < 0.05, **: *p* < 0.01, and ***: *p* < 0.001; colors indicate combination comparison to either GSH or PEF; ns: not significant). n.d.: not determined.

GSH is a known antioxidant used in all body cells to fine-tune oxidatively challenging situations and eustress [15]. Thus, we investigated whether the combination treatment would alter this redox balance based on changed levels of either oxidants (ROS) or antioxidants (GSH). GSH is the main source of replenishing the protein thiol pool, and total intracellular GSH was quantified using the thioltracker agent. Twenty-four hours after treatment, A375 (Figure 2A) and A431 (Figure 2B) cells showed a markedly enhanced intracellular GSH pool. However, at early time points after treatment, such as 0 h and 2 h, intracellular thiol pools were similar or decreased in combination treatments in A375 (Figure 2C), MNT-1 (Figure 2D), A431 (Figure 2E), and (except for 2 h) in SCC-25 (Figure 2F) cells. Similar results were seen with exposure of GSH alone in the absence of PEF, and the results were overall similar to GSH + PEF conditions. By contrast, PEF treatment alone left the intracellular GSH levels unchanged. Contrary to what we had expected, these results suggested that PEF treatment did not yield an intracellular GSH overload but did not influence GSH uptake within 2 h after PEF and GSH exposure. By contrast, at 24 h, intracellular GSH levels were elevated in GSH + PEF combination treatment, while GSH and PEF mono treatment did not affect cells considerably. 

**Figure 3:**
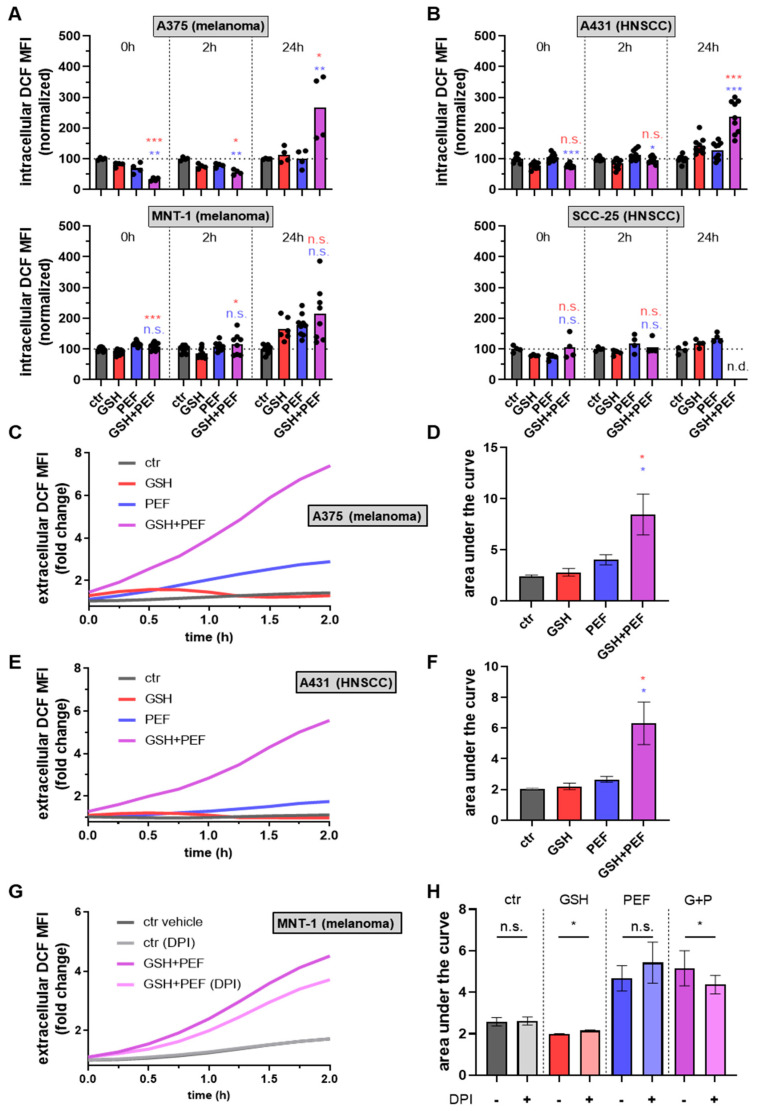
Intracellular ROS levels. (**A**,**B**) intracellular ROS levels measured using the pan-ROS dye CM-H_2_-DCF-DA in melanoma (A375, MNT-1; (**A**)) and SCC (A431, SCC-25; (**B**)) cells immediately (0 h), 2 h, and 24 h after exposure to the four treatment conditions and analysis by flow cytometry followed by normalization to control (ctr) conditions; (**C**–**F**) representative DCF fluorescence kinetic and quantification of resulting areas under the curve for A375 (**C**,**D**) and A431 (**E**,**F**) cells; (**G**,**H**) similar experiment as in C-F but using vehicle or DPI (NOX inhibitor) pre-treated MNT-1 cells and comparison of untreated and combined PEF + GSH-treated cells. Data show one representative (**C**,**E**,**G**) or mean of at least two independent experiments with several replicates (**dots**) each. Statistical analysis was performed using a *t*-test (**H**) or one-way analysis of variances with Turkey’s multiple comparison test (*: *p* < 0.05, **: *p* < 0.01 and ***: *p* < 0.001; colors indicate combination comparison to either GSH or PEF; ns: not significant). G+P: GSH + PEF treatment; n.d.: not determined.

**Figure 4:**
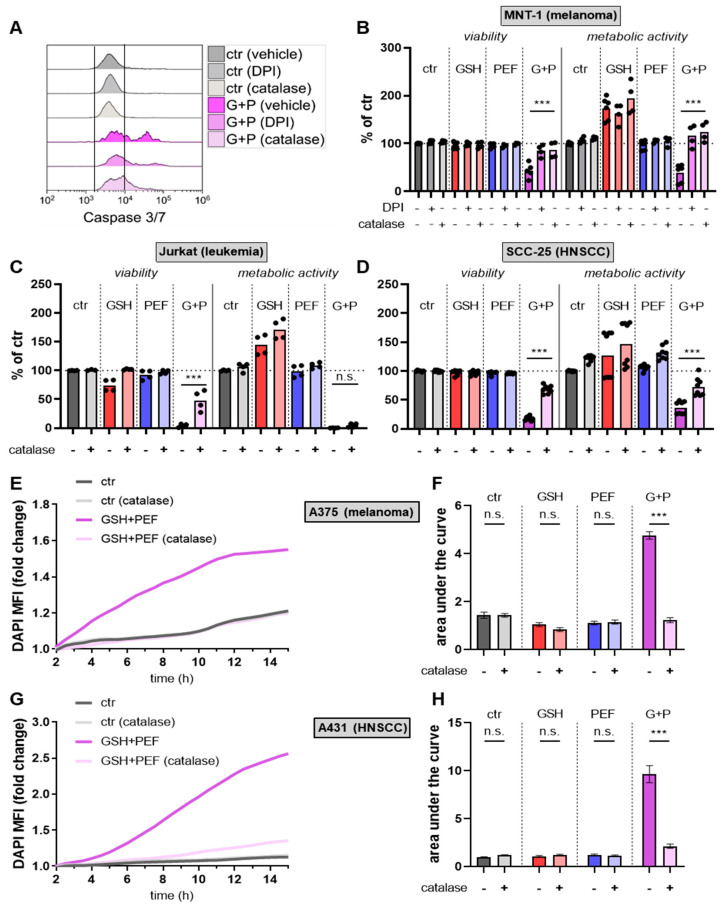
ROS dependence of combined GSH + PEF toxicity. (**A**) representative histograms of activated caspase 3/7 fluorescence in MNT-1 melanoma cells in the presence or absence of NOX inhibitor DPI or the H_2_O_2_-degrading enzyme catalase in untreated and combination-treated cells; (**B**–**D**) control (ctr)-normalized viability (as determined using flow cytometry) and metabolic activity (resazurin transformation) of MNT-1 (**B**), Jurkat (**C**), and SCC-25 (**D**) cells 24 h after exposure to mono and combination treatments in the presence or absence of DPI or catalase; (**E**–**H**) microplate reader-generated representative kinetic (**E**,**G**) and area under the curve quantification (**F**,**H**) of DAPI (terminally dead cells) of A375 (**E**,**F**) and A431 (**G**,**H**) cells following mono and combination treatment in the presence or absence of catalase. Data showed one representative (**A**,**E**,**G**) or meant of at least two independent experiments with several replicates each. Statistical analysis was performed using *t*-test (***: *p* < 0.001; ns: not significant). G+P: GSH + PEF treatment.

**Figure A2:**
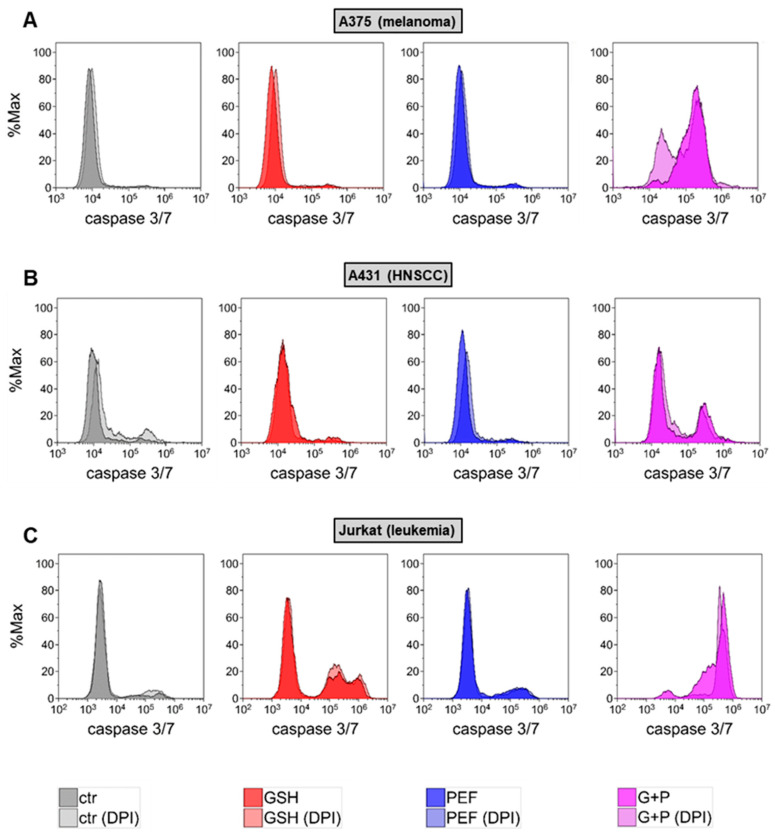
Cell viability of cells across all conditions in the presence or absence of DPI. (**A**–**C**) representative overlay histograms of activated caspase 3/7 fluorescence in A375 (**A**), A431 (**B**), and Jurkat (**C**) in the presence or absence of NOX inhibitor DPI in untreated and combination-treated cells at 24 h.

**Figure A3:**
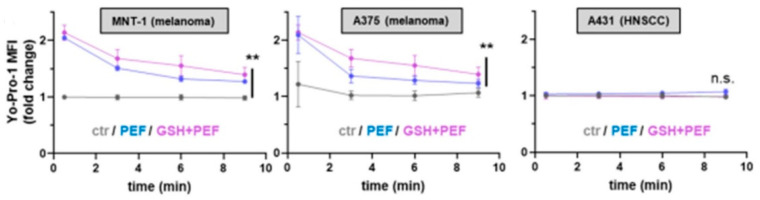
Altered cell permeabilization and GSH aggregation. YO-PRO1 uptake kinetic of PEF and PEF + GSH treated MNT-1, A375, and A431 cells immediately to up to 10 min following treatment, and normalization to values of untreated cells (ctr). **: *p* < 0.01.

Changes in the presence or abundance of GSH can be hypothesized to affect reactive oxygen species (ROS) production or levels. Therefore, intracellular ROS were measured at the same time points as intracellular GSH levels. Interestingly, in A375 (Figure 3A) and A431 (Figure 3B) skin cancer cells, a decline in intracellular ROS was also observed in combination regimens over mono treatments. Also similar to intracellular GSH levels, there was a strong increase in steady-state intracellular ROS levels at 24 h following GSH + PEF combination exposure, while mono treatments did not show much effect. These results suggested a massive perturbation of redox homeostasis in GSH + PEF-treated skin cancer cells with diverging trends between short- and long-term analysis. Most cell types possess NADPH oxidases (NOX), many of them also at cell membranes, even if in low quantities. NOXs can produce ROS (superoxide) in the extracellular space. We investigated the possibility of extracellular ROS accumulation following mono and combination treatments by performing kinetic measurements in A375 (Figure 3C,D) and A431 (Figure 3E,F) cells. Indeed, GSH + PEF combination treatment generated significantly enhanced levels of ROS compared to mono treatments in both skin cancer cell lines. Results were, in principle, similar in MNT-1 cells (Figure 3G,H), albeit PEF alone already increased ROS strongly in these cells. Interestingly, DPI, an inhibitor of NOX enzymes, significantly reduced extracellular ROS production after combination treatment. Nevertheless, there must be other ROS sources at play, as the amplitude of the DPI-induced ROS reduction was modest. Notwithstanding this, we next sought to investigate the role of ROS in the GSH + PEF-induced apoptosis-induction by pre-incubating cells with DPI or extracellular catalase, an hydrogen peroxide (H_2_O_2_)-depleting enzyme (Figure 4A). To our surprise, DPI and catalase fully abrogated combination treatment-mediated cytotoxicity and reduced metabolic activity in MNT-1 cells (Figure 4B). In A375 (Figure A2A), A431 (Figure A2B), and Jurkat (Figure A2C), DPI-mediated cytotoxicity reduction was less pronounced but significant in the case of catalase in the latter cell type (Figure 4C) as well as in SCC-25 cells (Figure 4D). These findings strongly suggested a role of ROS as a mechanism of GSH + PEF-induced cancer cell decline. Finally, we were interested in understanding the cell death kinetics of the GSH + PEF combination treatment. To this end, we performed kinetic measurements on terminally dead (DAPI-positive) dead cells in A375 (Figure 4E,F) and A431 (Figure 4G,H) cells. Cell death was continuous and peaked at 12 h post-treatment in A375 and >14 h in A431 cells. In A375 but not A431, this may potentially be linked to a significantly enhanced electroporation in the cells with PEF in the presence of GSH (Figure A3). Strikingly, pre-treatment of both cell types with catalase abrogated cell death induction in GSH + PEF combined treatments to baseline levels, ultimately leading to a significantly reduced cytotoxicity in A375 (Figure 4F) and A431 (Figure 4H) skin cancer cells. 

## 3. Discussion

Electrochemotherapy (ECT) is an established oncological treatment scheme, especially in patients with palliative skin cancer (malignant melanoma, squamous cell carcinoma) [16,17]. While bleomycin and cisplatin are established cytostatic drugs in ECT, non-responding patients need alternative treatment options. We here provide compelling in vitro evidence that supraphysiological levels of extracellular glutathione (GSH) synergistically combined with pulsed electric field (PEF) treatment to exert cytotoxic effects in six cancer cell lines.

By using two cell lines of three cancer types (malignant melanoma, squamous cell carcinoma, leukemia), we could compare the efficacy of our approach and did not identify a major dependency of PEF + GSH combination treatment on tumor type. This could be owed to two reasons. First, GSH is a universally important molecule in all body cell types, including non-nucleated ones [18]. Second, PEF, especially when applied in the microsecond range, affects cell membranes by inducing membrane charging [19] and pore formation [20]. The basic structure of lipid bilayer cell membranes is similar among different cell types, apart from alterations in, e.g., cholesterol content, lipoproteins, and lipid rafts [21]. In addition, it was suggested that intracellular membranes (e.g., ER) could also be affected by microsecond PEF [22]. However, it should be noted that PEF treatment alone had no notable effect in any of the cell lines or assays investigated. Hence, the key question is the contribution of GSH in this combination treatment. One possibility is that GSH someone extends the opening of the cell membrane pores by interacting with the proteins near the pore site, thereby allowing extensive amounts of cytosol to exit to cell and thereby inducing cell death. This idea is contrasted by our findings that GSH + PEF combination treatment cell death takes place slowly and continuously over many hours and involves activation of caspases 3 and 7, arguing for regulated (apoptotic) cell death rather than necrosis [23]. A second possibility is that PEF treatment induces the formation of GSH aggregates, as known from its oxidized form, GSSG containing two oxidized GSH molecules [24]. Much larger GSH aggregates are observable, e.g., under extensive heat-stress conditions [25]. It is known that protein aggregates can be cytotoxic to cells [26,27].

The strongest association we found with GSH + PEF combination toxicity was the involvement of ROS. ROS are generated upon PEF treatment [28]. In our study, PEF treatment alone did not promote intracellular ROS release in flow cytometry but in three cell lines when measured kinetically. Since DPI, a known inhibitor of NOX in vitro and in vivo [29], reduced ROS release only to a modest extent, other sources, such as mitochondrial ROS due to, e.g., PEF-induced hyperpolarization, may be involved [30]. Since GSH and protein thiols are known to be consumed to deteriorate ROS [31], this could explain the decrease of intracellular GSH 0 h and 2 h after GSH + PEF treatment because GSH is consumed. At the same time, the question is why the abundant extracellular GSH is not transported into the cell to re-establish homeostatic levels. It is important to note that a major pathway of intracellular GSH re-generation is through the import of cystine through the chloride-dependent cystine/glutamate antiporter called solute carrier family 7 (SLC7A11) [32]. Potentially, the transporter activity is decreased through PEF treatment, or the limiting step of GSH intracellular re-constitution is the availability of extracellular cystine. Direct shuttling of GSH across membranes is known for mitochondrial membranes via SLC25A39 [33] but has been less described for cell membranes. In addition, the question arises whether PEF-treated GSH had lost its antioxidative activity. This could be assumed since excess GSH protects tumor cells from cell death [34]. Such protection was not the case since, in our study, tumor cells elaborately died following combination treatment. Therefore, it can be assumed that cell death-inducing stimuli were either too strong or the antioxidant effect of GSH was insufficient to abrogate cell demise, or GSH itself was deteriorated or complexed. A former study found increased GSH antioxidant activity following PEF treatment with about ten to twenty-fold higher field strengths than our study setup, for which no changes were reported [35]. Hence, increased antioxidant activity of PEF-treated GSH unlikely explains the nature of our results.

## 4. Materials and Methods

### 4.1. Cell Culture

Six human cell lines were used in this study, namely MNT-1 melanoma cells (ATCC: CRL-3450), A375 melanoma cells (ATCC: CRL-3224), A431 cutaneous cell carcinoma cells (ATCC: CRL-1555), SCC-25 squamous cell carcinoma cells (ATCC: CRL-1628), Jurkat lymphoma cells (ACC282), and THP-1 acute myeloid leukemia cells (ATCC: TIB-202). The skin cancer cells were cultured in Dulbecco’s Modified Eagles Medium (DMEM; Pan-Biotech, Aidenbach, Germany) that was supplemented with 1% penicillin/streptomycin, 1% glutamine, and 10% fetal bovine serum (all Sigma-Aldrich, Taufkirchen, Germany) and the leukemia cell lines in Roswell Park Memorial Institute (RPMI; Pan-Biotech, Aidenbach, Germany) with the same supplements.

### 4.2. Pulsed Electric Fields (PEF) Application

An electro square porator (ECM 830; BTX Havard Apparatus, Holliston, MA, USA) was used for applying pulsed electric fields (PEF). PEF pulse duration was 100 µs at 1 Hz and 0.5–0.75 kV/cm as compliant with the European standard operating procedures for electrochemotherapy [36]. Before PEF treatment, cells received either vehicle controls or 10 mM Glutathione (Sigma-Aldrich, St. Louis, MO, USA). In some experiments, cells were pre-incubated with diphenyleneiodonium (DPI, 1 µM; Sigma-Aldrich, St. Louis, MO, USA), being a general NADPH oxidase (NOX) inhibitor or the hydrogen peroxide (H_2_O_2_)-degrading enzyme catalase (20 µg/mL, Sigma-Aldrich, St. Louis, MO, USA). For the PEF treatment, the cells were transferred to electroporation cuvettes (2 mm; Biozym Scientific, Oldendorf, Germany). Control samples were transferred to such cuvettes as well as mock treatment. Afterward, cells were added to microwell plates or immediately used for downstream analysis. To confirm electroporation, YO-PRO-1 (0.5 µM; Thermo Fisher Scientific, Waltham, MA, USA) was added to the cells for 15 min at a pre-determined frequency (immediately, 3 min, 6 min, and 9 min after treatment). Subsequently, the YOPRO-1 mean fluorescence intensities were measured using flow cytometry and normalized.

### 4.3. Metabolic Activity

To identify the cells’ metabolic activity, resazurin (100 µM; Alfa Aesar, Kandel, Germany) was added 20 h after their treatment. Then, the cells were incubated for 4 additional hours. During this time, resazurin is transformed by metabolically active cells into fluorescent resofurin, which can be quantified in the cells’ supernatants. This was accomplished using a multimode plate reader (F200; Tecan, Männedorf, Switzerland) at λ_ex_ 560 nm and λ_em_ 590 nm. Data were normalized to controls.

### 4.4. Cell Viability

Cell viability was determined by incubating the cells for 30 min in the incubator with CellEvent caspase 3/7 reagent (1 µM; Thermo Fisher Scientific, Dreieich, Germany) and 4′, 6-Diamidino-2-phenylindole dihydrochloride (DAPI, 1 µM; BioLegend, Amsterdam, The Netherlands). Before, adherent cell lines were detached using accutase (BioLegend), while suspension cell lines were collected directly into round-bottom 96-well plates. Cells were acquired using flow cytometry (CytoFLEX LX; Beckman-Coulter, Krefeld, Germany). For viability analysis, the percentage of the caspase 3/7-negative, DAPI-negative population was quantified among all intact cells gated from the forward and side scatter dot plots. Data were normalized to controls. Alternatively, cell death was quantified dynamically. For this, cells were seeded in 96-well plates after treatment, and DAPI was added. Then, the plates were added to a pre-heated and pre-gassed microplate reader (M200; Tecan) equipped with temperature control and CO_2_ supply units (Tecan, Männedorf, Switzerland). In addition, the outer wells of the well plates were filled with deionized water to protect the inner wells from evaporation during the extended 12 h-incubation period in the microplate reader and DAPI fluorescence acquisition (λ_ex_ 365 nm and λ_em_ 450 nm) every 30 min. Data were normalized to individual wells’ baseline values at 2 h after the plate acquired the correct temperature. Data analysis was performed by calculating the resulting areas under the curve (AUC).

### 4.5. Intracellular Glutathione

To determine the intracellular glutathione content, the cells were stained with propidium iodide (PI; 5 µg/mL; Santa Cruz Biotechnology, Heidelberg, Germany) to discriminate live from dead cells, and ThiolTracker Violet (10 µM; Thermo Fisher Scientific) in PBS containing calcium and magnesium (Pan-Biotech). The staining was done for 30 min in the incubator. During this time, the dye penetrates the cell membranes and binds free protein thiols (reduced GSH) in all cellular compartments. The binding leads to a conformational change and fluorescent properties (λ_ex_ 405 nm and λ_em_ 525 nm). After the staining, the cells were washed with fully supplemented cell culture medium and resuspended in FACS buffer for acquisition by flow cytometry (CytoFLEX S; Beckman-Coulter). This process was done for cells immediately after treatment and after 2 h and 24 h incubation. Data were normalized to control samples.

### 4.6. Intracellular and Extracellular Reactive Oxygen Species

To assess the intracellular oxidation, the cells were stained with DAPI to exclude dead cells and CM-H_2_DCFDA (1 µM; Thermo Fisher Scientific) for cytosolic reactive oxygen species (ROS). The staining was performed for 15 min in the incubator in PBS. Afterward, the cells were washed, resuspended in fully supplemented medium, and measured using flow cytometry (CytoFLEX S). This was done immediately and 2 h and 24 h after treatment. To dynamically assess the production or release of ROS into extracellular space, the cells were exposed to the different PEF treatment modalities, added to a 96-well plate containing DCFH, and put into a 37 °C-pre-heated and pre-CO_2_-gassed microplate reader (F200) to dynamically assess fluorescence intensities every 15 min (λ_ex_ 485 nm and λ_em_ 525 nm). The outer wells of the plate were filled with deionized water to protect from evaporation.

### 4.7. Software and Statistical Analysis

Flow cytometry samples were quantitively analyzed in this study using Kaluza analysis software 2.1.3 (Beckman-Coulter). Data normalization was done using Excel 2021 (Microsoft, Redmond, WA, USA). Data analysis and graphing were done utilizing prism 9.4.1 (GraphPad Software, San Diego, CA, USA). One-way ANOVA with Turkey’s multiple comparison test or *t*-test was executed to determine the degree of statistical significance between groups. The level of significance is indicated as follows: *p* < 0.05 (*), *p* < 0.01 (**), and *p* < 0.001 (***).

## 5. Conclusions

This first-time reporting of extensive cytotoxic effects of extracellular GSH across several PEF-treated cancer cell types could lead to identifying a new molecule to be used within local ECT injection schemes in palliative oncology. The benefits of GSH are the absence of toxicity due to decades of experience as a dietary nutrient and its role as an intracellular oxidant, making this “endogenous drug” a promising candidate to be tested in animal models for the safety and efficacy of the treatment when benchmarked against standard ECT regimes using cisplatin or bleomycin. Future studies also need to elaborate the relationship between electric field intensity and cytotoxic GSH activity for different intensities, concentrations, time points, and cell lines. Another striking point that needs to be investigated using cells and tissue models is whether the antioxidant activity of GSH is altered due to PEF processing.

## Figures and Tables

**Table 1:** Coefficient of drug interaction (CDI). CDI was calculated as follows: CDI = (AB)/(AxB). AB is the ratio of the combined treatment group to the control group, and A or B is the ratio of the mono-treatment group to the control group. Therefore, CDI < 1 indicates synergism, CDI < 0.7 indicates significant synergism, CDI = 1 indicates an additive effect, and CDI > 1 indicates an antagonistic effect. The CDI was determined for both cell viability and metabolic activity. n.d. = not determined.

	A375	MNT-1	A431	SSC-25	THP-1	Jurkat
cell viability	0.08	0.56	0.33	0.12	0.56	0.04
metabolic activity	0.09	0.29	0.16	0.55	0.05	0/n.d.

## Data Availability

Data can be retrieved from the corresponding author upon reasonable request.
